# A tool for simulating collision probabilities of animals with marine renewable energy devices

**DOI:** 10.1371/journal.pone.0188780

**Published:** 2017-11-29

**Authors:** Pál Schmitt, Ross Culloch, Lilian Lieber, Sverker Molander, Linus Hammar, Louise Kregting

**Affiliations:** 1 Marine Research Group, Queen’s University Belfast, Belfast, Northern Ireland, United Kingdom; 2 Environmental Systems Analysis, Energy and Environment, Chalmers University of Technology, Gothenburg, Sweden; 3 Swedish Agency for Marine and Water Management, Gothenburg, Sweden; Centro de Investigacion Cientifica y de Educacion Superior de Ensenada Division de Fisica Aplicada, MEXICO

## Abstract

The mathematical problem of establishing a collision probability distribution is often not trivial. The shape and motion of the animal as well as of the the device must be evaluated in a four-dimensional space (3D motion over time). Earlier work on wind and tidal turbines was limited to a simplified two-dimensional representation, which cannot be applied to many new structures. We present a numerical algorithm to obtain such probability distributions using transient, three-dimensional numerical simulations. The method is demonstrated using a sub-surface tidal kite as an example. Necessary pre- and post-processing of the data created by the model is explained, numerical details and potential issues and limitations in the application of resulting probability distributions are highlighted.

## Introduction

The probability of collisions between mobile marine fauna and fixed or moving parts of MRE devices might be relevant for the further development of the MRE sector, and has gathered interest from the scientific community, consultants, regulators and device developers [1, 2]. Developers have been tasked by regulators to perform continuous monitoring and mitigation measures, including the shut-down of the device if marine megafauna is observed within the vicinity, as was the case for SeaGen, the world’s first tidal turbine to be fully commissioned as a grid connected power plant [3]. These shut-down measures provide safety for the animals, but also result in a lack of observations of animal-device interactions [4]. Since the technology has huge potential and is expected to be deployed across the world [5] in the coming years, increasing the understanding of collision risks is of high importance.

The problem of establishing the overall collision risk for an installation in a given location is dependent on both physical and biological factors. Here, collision probability is represented by the probability of co-occurrence of a MRE-device with an animal simplified to a to a three-dimensional ellipsoid shape. The application of a 4D simulation (a three dimensional representation of the MRE-device and an animal over time) is purely based on geometry and kinematics, which should be defined using biological data, possibly observed in the field [6–8]. Avoidance behaviour of the animal could be used to define the animal trajectory in the simulation or the probability of the existence of an animal close to the device [9–14] could be included in postprocessing to derive a collision risk estimate, which could then be incorporated into an environmental risk assessment.

Several studies [15, 16] of horizontal axis turbine collision risks have applied the same formula to estimate the collision probability in the rotor-swept area. The blades of a horizontal axis tidal turbine sweep a disc-shaped volume, which is simplified to a circular plane. The collision probability *P*_*C*_ can then be expressed as

$$\begin{array}{c} P_{C}=\frac{N\omega L\text{cos}(\alpha )}{v} \end{array}$$ (1)

with *ω* being the rotational velocity, *N* the number of blades, *v* the velocity and *α* the angle of an animal of length *L* relative to the flow direction. The conceptual model of this collision probability formulation is that the percentage of the entire disc area swept by the blades during the time an animal passes, is a measure for the mean collision probability across the disc.

This does not provide any information on the distribution of the probability across the disc area. For a standard horizontal axis turbine, the probability of impact at the axis is one and decreases towards the blade tips. Close to the axis, the distance between blades could be less than the width of the passing animal, so the collision probability would also be one. The accuracy of this formulation for specific cases will thus depend on the shape of the turbine and relative size and shape of the animal. It should also be noted that besides the collision probability, additional data might be of interest when assessing a collision, for example, the relative velocity or shape, stiffness and material properties of the structure [27].

The work presented in this paper is motivated by the need to find an equivalent value to Eq 1 for other types of tidal turbines and wave energy converters with more complex shapes and forms of motion than a horizontal axis turbine, for which currently no solution exists. For example, flap type wave energy converters move back and forth in the water column as a single [17] or an array of structures [18]. The ORPC device is a helical cross flow tidal turbine [19] and as such, an animal might encounter moving blades more than once during passage of the enclosed volume, even if the simplest case of an animal following a straight path is assumed. It should also be noted that Eq 1 implies animal motion perpendicular to the plane.

Literature on mathematical modelling of collision probability distributions is limited and applications vary considerably from one area of application to another. For example, the collision probability of space craft or satellites with space debris has been investigated in great detail, typically by simulating an object’s flight path and evaluating the minimum distance [21, 22], sometimes even taking into account possible evasion manoeuvres or uncertainty of parameters such as shape and size of motion paths [23]. Due to the large number of objects involved, massive parallel computations are now being applied [24]. In another example, a mathematical model was developed to investigate the collision probability and minimum safe overtaking distance of two vessels at sea [25]. This model was used to derive a safe overtaking distance dependent on overtaking speed and further vessel characteristics like the radius of turn and response time. An investigation of whether an optimum speed exists to minimize the amount of rain falling onto a moving body found, even for simple shaped bodies like cylinders or parallelepipeds, that the solution depended on rain/wind direction and the shape of the body [26]. Raindrops were represented as a flux, so the actual size of the drops was not taken into account and the method was applied to bodies moving with constant velocity on a straight line.

**Fig 1:**
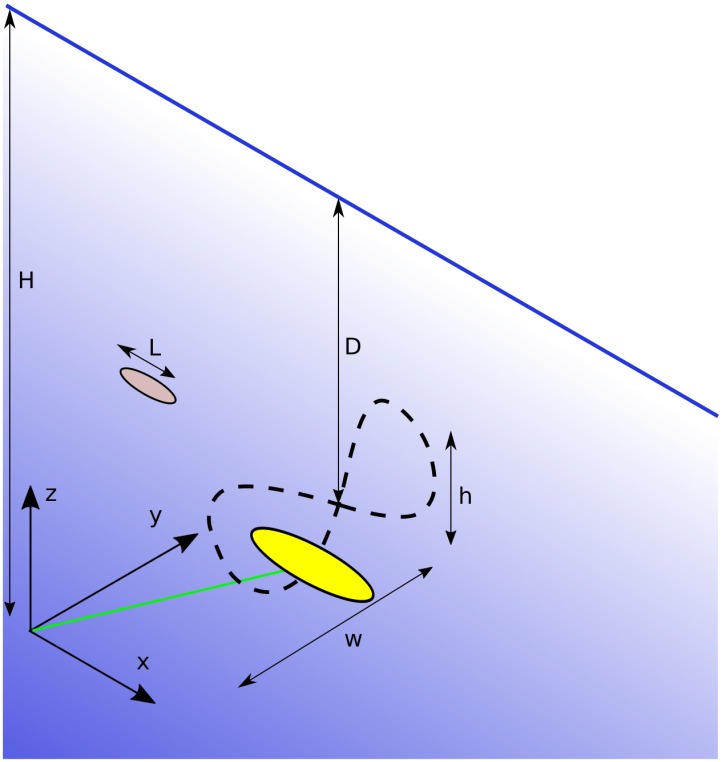
Schematic illustration of the tidal kite (yellow), tether (green) and flightpath (dashed line) with main variables as defined in Table 1 and coordinate system as used in the simulations. The grey elliptic symbol represents the animal under risk of collision. The foundation is located at the origin.

The device used for a demonstration of the method is modelled on the Deep Green sub-sea tidal kite developed by Minesto [20]. The kite is tethered to the sea floor and follows a trajectory similar to a figure of eight in the water column, operating downstream of the foundation, see Fig 1. Therefore, in this example, an animal might initially pass one component of the structure (e.g. at the front of a cross flow turbine), but further down its trajectory, later in time, it might still collide. The kite is an interesting case because it is not covered by any method presented to date and collisions with the wing might have different consequences from collisions with the tether, since material properties vary significantly.

While the definition of a collision (zero or negative distance between two shapes) is identical for all cases discussed, the cases of interest in the present study are different. Firstly, compared to collision models of space craft or ships, which are typically described by convex, closed surfaces, the DeepGreen sub-sea kite (and many other marine structures of interest) consists of a complex shape, providing opportunities for collisions on multiple surfaces at every point in time. Secondly, the final result is not a collision probability for an object on a given course but a distribution of collision probabilities over the entire area covered by the device. The most general solution to obtain a probability distribution for collisions is to simulate all motions in three-dimensional space over time, thus four dimensions. In theory any geometric shape could be moved along any path of motion. This paper presents details of such an implementation and an exemplary application to a hypothetical animal, loosely modelled on a seal. The kite dimensions, trajectory and speed are inspired by the Minesto quarter-scale device undergoing field testing in a tidal channel in Strangford Lough, Northern Ireland, UK.

## Methods

The numerical model was implemented in the open source freeCAD toolbox [28], the source code is provided in S1 File. freeCAD is a general purpose Computer Aided Design framework and allows the user to create and manipulate geometric objects via scripting in the python programming language or a graphical user interface. The main functions used in the simulations are rotations and translations to position objects at each time step and tools to evaluate the distance between two shapes.

The kite geometry is assumed to be identical for all simulations in this paper and defined as two parts, the tether and the kite, see Fig 1. The later allows the user to record collisions with the tether and the kite separately, such that the consequences of a collision might be evaluated taking the structural properties at the impact location into account.

The kite has a span width of 3*m* and the length of the tether is 25*m*.

Each simulation is defined by the following set of variables:

- animal length (*L*)
- animal velocity (*v*)
- the animal’s initial position (*z*, *y*)
- a phase lag (*δ*) between animal and kite motion
- the time it takes the kite to fly the entire track (*T*)
- the mean kite depth (*D*)
- the water depth (*H*)
- the height (*h*) and width (*w*) of the figure of eight describing the kite trajectory

For the simulations presented here, a parametric flight path, loosely modelled on the Minesto quarter-scale device, was implemented. Animals were represented by ellipsoids, with the major radius set to half the animal length and the minor radii set to a third of the animal length. The animals were set to move with a constant velocity in positive x direction.

For each time step the following tasks are performed:

- The kite is moved into its new position by a sequence of three rotations around the bottom joint:
  1. *α* is obtained from the previous two kite positions to ensure the kite is pointing in the direction of the flight path.
  2. *β* turns the device, such that the centre of the kite oscillates around the mean depth with a period of *T*/2
    $$\begin{array}{c} \beta =\arccos(\frac{H-D+\frac{h}{2}\sin\left(\frac{\pi }{T}\left(t-\delta \right)\right)}{L}) \end{array}$$ (2)
  3. *δ* positions the kite at a y position, oscillating around the center axis with period *T*.
    $$\begin{array}{c} \gamma =\arcsin(\frac{\frac{w}{2}}{\sin\left(\beta \right)L\sin(\frac{2\pi }{T}\left(t-\delta \right))}) \end{array}$$ (3)
- The seal position in *x* direction is next updated from its initial position (0, *y*, *z*) according to linear translation:
  $$\begin{array}{c} x(t)=v\cdot t \end{array}$$ (4)
- The minimum distance between the animal and the kite or tether is then evaluated. Any distance below a set threshold (1*mm* in this case) is considered a collision and the simulation is stopped.
- If no collision occurs, the simulation continues until the animal passes the maximum downstream kite position, defined as the length of the tether plus the height of the kite structure.

The occurrence of a collision also depends on the phase relation between animal and device. Therefore, a phase lag was used to vary the starting position of the kite. Simulations with varying phase lags must be run for all animal positions, animal sizes, velocities and kite parameters. Establishing probabilities requires considerable numbers of runs. 50 delays distributed evenly over the kite period provide probabilities with an accuracy of 2%.

To obtain a collision probability distribution across the device, 21 different depths and 15 positions across the width of the flight path were simulated, creating a 1*m* × 1*m* grid. With 50 delays, *NSim* = 15750 simulations must be run to obtain a single probability distribution for one configuration of device and animal. If different operating conditions of the device are of interest, the previous number would again increase by that factor, creating a considerable computational burden. Since simulations are independent of each other, parallelisation can easily be achieved. Runtime scales almost linearly with time step and a full set of 15000 simulations took approximately 24h on 28 cores on a AMD Opteron(TM) Processor 6272.

To demonstrate the application of this numerical model, we present a time step convergence study and then three physically plausible changes in operating conditions of the MRE device.

All cases presented use the settings given in Table 1.

**Table 1:** Parameters used for the baseline case. The midpoint of the flightpath is at *y* = 0*m*. Lists are defined as start:increment:final value.

*L*	*z*	*y*	*v*	*δ*	*T*	*H*	*D*	*h*	*w*
[*m*]	[*m*]	[*m*]	[*m*/*s*]	[*s*]	[*s*]	[*m*]	[*m*]	[*m*]	[*m*]
1.410	0:1:20	-7:1:7	1.8	0:T/50:T	8	20	7	3	10

### Probability metrics

The collision probability *P*_*A*_ across the entire investigated cross-section *A* is defined as

$$\begin{array}{c} P_{A}=\frac{NColl}{NSim} \end{array}$$ (5)

with *NSim* the number of all simulations and *NColl* the total number of collisions.

The collision probability as defined in Eq (1) refers only to the area swept by the turbine blades. As many novel MRE devices cannot be compared to conventional horizontal axis turbines, we define a second collision probability as follows:

$$\begin{array}{c} P_{SweptA}=\frac{NColl}{NCollPos} \end{array}$$ (6)

where *NCollPos* is the number of positions at which at least one collision occurred for all delays tested. This value is directly comparable to the one obtained in Eq (1) but for the MRE device investigated in this paper *NCollPos* is a function of the MRE device configuration. The post processing of the data generated by freeCAD was implemented in R [29].

### Time step convergence

The temporal resolution of the simulation must be chosen appropriately by performing a convergence study. The baseline case simulations were run for decreasing time step size, beginning with 0.2*s*. Table 2 shows the number of collisions *NColl* over time step size. It can be observed that the number of collisions varies for time steps larger then 0.1*s* for the tether *NColl*_*Tether*_ and 0.05*s* for the kite *NColl*_*Kite*_. In some very rare instances, the animal hits the kite and tether simultaneously (*NColl*_*Both*_), but although those numbers still show variations for time steps of less than 0.1*s* they seem negligible for the collision probabilities between the animal and the MRE device. The slower convergence of the kite seems plausible, given the higher absolute velocity of its motion.

**Table 2:** Total number of collisions with varying timestep for the baseline case (*h* = 3*m*, *T* = 8*s*, *D* = 7*m*).

	0.2*s*	0.1*s*	0.05*s*	0.01*s*
*NColl*_*Kite*_	531	594	581	581
*NColl*_*Tether*_	935	970	970	970
*NColl*_*Both*_	52	54	34	34
*NColl*	1413	1510	1517	1517
*P*_*A*, *Kite*_	3.37	3.77	3.69	3.69
*P*_*A*, *Tether*_	5.93	6.16	6.16	6.16
*P*_*A*, *Device*_	8.97	9.59	9.63	9.63

The probabilities obtained for a time step of 0.1*s* seem accurate enough for this application, so in all following cases this value was used. It should be noted however, that the adequate time step will always be a function of animal and kite velocity, so might vary for different simulations. We recommend that the highest velocity case is used to assess suitable parameters.

## 1 Results

### Case 1: Baseline case

Fig 2.1 presents results for the baseline case. At each position across the investigated cross-section the probability of a collision is evaluated and displayed in a coloured and scaled circle. Overall, the area where collisions occur is approximately V shaped. The only position where a collision is inevitable is the mooring point at the bottom, where the tether maintains a fixed position, blocking the passage at all times. Above the mooring point, collision probabilities decrease to approximately 40% in the centre and continue to decrease with increasing distance from the centreline. This is due to the fact that the tether passes twice through the middle section during each period. Around the mean flight depth of the kite, the probability of a collision increases again to up to 80% in the centre. For the baseline case *P*_*A*_ is 9.59%, while *P*_*SweptA*_ was 25.81%. The majority of collisions are with the tether, the ratio *NColl*_*Tether*_/*NColl*_*Kite*_ is 1.7, Table 3.

**Fig 2:**
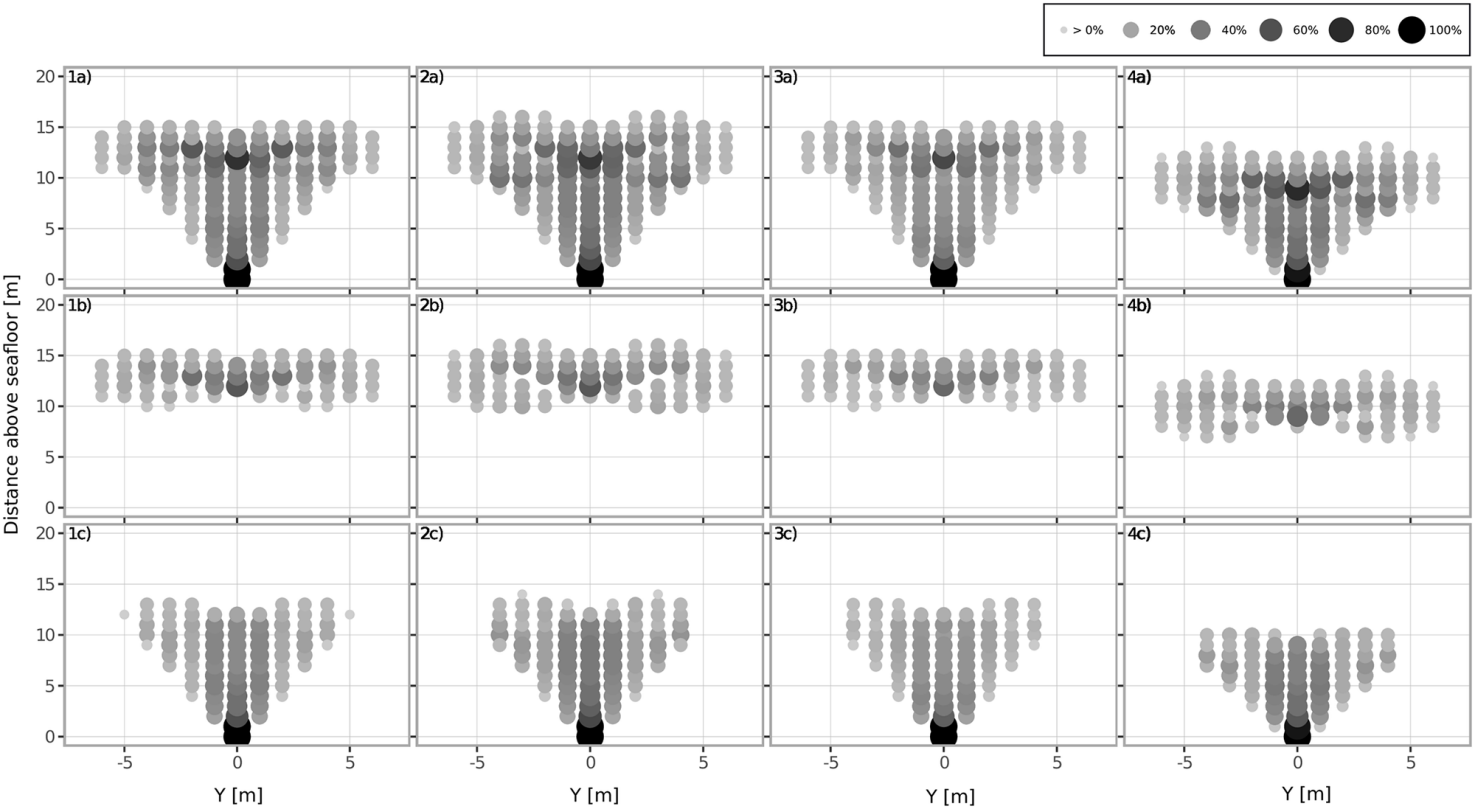
Probability (%) of a collision at each position in the cross-section between an animal and the a) entire structure, b) the kite, only and c) the tether, only for different configurations. Columns represent the four different cases described in the main text; 1) baseline, 2) variation of kite trajectory, 3) longer kite period and 4) increased flight depth.

**Table 3:** Number of collisions *NColl* and probabilities *P* as defined in the text above for varying configurations, based on 15750 simulations. The parameter changed from the baseline case is set in bold.

	Case 1 (Baseline)	Case 2	Case 3	Case 4
*h* [*m*]	3	**4**	3	3
*T* [*s*]	8	8	**10**	8
*D* [*m*]	7	7	7	**10**
*NColl*_*Kite*_	540	656	462	563
*NColl*_*Tether*_	916	1023	826	815
*NColl*_*Device*_	54	39	28	30
*NColl*	1510	1718	1316	1408
*NColl*_*Tether*_/*NColl*_*Kite*_	1.7	1.6	1.8	1.4
*NCollPos*	117	128	117	111
*P*_*A*, *Kite*_	3.77	4.41	3.11	3.77
*P*_*A*, *Tether*_	6.16	6.74	5.42	5.37
*P*_*A*, *Device*_	9.59	10.91	8.36	8.94
*P*_*SweptA*, *Device*_	25.81	26.84	22.5	25.37

### Case 2: Variation of kite trajectory

Fig 2.2 shows results for a case with *h* = 4*m*. With the kite flying a higher figure of eight, no collisions occur with the kite in a small region off the centre, around *y* = ±2 − ±3. The main difference to the baseline case is nevertheless the increase in the number of total kite collisions from 540 to 656. As expected, the overall swept area increases in the vertical plane from approximately 5*m* to 8*m*, the number of positions where collisions occur increases to 128, Table 3. Overall, the total collision probability in the total area increases slightly to 10.91% and the collision probability in the now larger swept area remains almost identical to the baseline case at 26.84%.

### Case 3: Longer kite period

When the same kite trajectory as in the baseline case is used but the kite period is increased from 8 to 10*s*, the overall collision distributions hardly seem to change, Fig 2.3. The collision probability relating to the total area as well as the one relating to the swept area decreases to the lowest value of all cases investigated with 8.36% and 22.5%, respectively.

### Case 4: Increased flight depth

Increasing the mean flight depth of the kite from 7*m* to 10*m* scales the collision distributions vertically, Fig 2.4. No collisions occur above 13*m* and the number of positions where collisions occur reduces to 111 while the probability of a collision across the swept area remains almost equal (25.37% *vs* 25.81%). The overall collision probability across the cross-section decreases slightly compared to the baseline case (8.94% *vs* 9.59%). Also, there was a notable decrease in the ratio between kite and tether collisions to 1.4.

## Discussion and conclusion

We present a numerical method to compute the spatial collision probability distribution for a novel MRE device. The method and processing is generally suited for probabilistic collision modeling of any structure and not necessarily limited to the marine environment, as long as geometries and motions are known. Care must be taken to choose appropriate temporal resolution and a convergence study should be carried out to choose an appropriate time step. The collision probability value obtained across the entire cross-section is somewhat arbitrary, but allows us to compare the outcome of different configurations of the MRE device in a given position. The collision probability evaluated over the swept area can be used instead of Eq 1 for novel MRE devices. Both probabilities show relatively little sensitivity to varying device parameters within physically reasonable bounds.

For each device configuration, a large number of positions exist, where (for straight, translational animal motion) no collision can possibly occur. Those positions could be established after completing the first kite trajectory and removed, which would reduce the computational time required for future simulations. Some care must be taken when using the results of Eqs 5 or 6 instead of Eq 1, otherwise the collision probabilities could be exaggerated.

In future applications the resulting collision probabilities could be weighted according to whether a collision is considered more severe, for example if certain parts of the structure (e.g. the rotor) are involved or if it occurs with higher relative velocities. Similar approaches have been discussed by [27].

Ecological data such as depth distributions and transit rates could be multiplied with the collision probability distribution to progress towards a more realistic estimate for collision risk. Arguably, the lack of knowledge on actual probability distributions for a certain species to occur in a given time or space and the reaction in the vicinity of a device are larger sources of error than the expected variations in collision probability due to design or operation of the MRE device.

## Supporting information

S1 File S1_File.fcmacro. (FCMACRO)

## References

[1] Wilson B, Batty RS, Daunt F, Carter C. Collision risks between marine renewable energy devices and mammals, fish and diving birds. Report to the Scottish ExecutiveScottish Association for Marine Science, Oban, Scotland, PA37 1QA. 2007; p. 1–105.

[2] Wood J, Joy R, Sparling C. Harbor Seal Tidal Turbine Collision Risk Models. An Assessment of Sensitivities. Prepared for PNNL / DOE by SMRU Consulting. 2015.

[3] Savidge G, Ainsworth D, Bearhop S, Christen N, Elsaesser B, Fortune F, et al Strangford Lough and the SeaGen Tidal Turbine In: Shields MA, Payne AIL, editors. Marine Renewable Energy Technology and Environmental Interactions, Humanity and the Sea. Springer Netherlands; 2014 p. 153–172. Available from: http://link.springer.com/10.1007/978-94-017-8002-5.

[4] Keenan G, Sparling C, Williams H, Fortune F. SeaGen Environmental Monitoring Programme Final Report. 10 Bernard Street Leith Edinburgh EH6 6PP United Kingdom +44 131 555 0506: Marine Current Turbines; 2011.

[5] Available from: http://www.4coffshore.com/windfarms/tidals.aspx.

[6] Hastie GD, Gillespie DM, Gordon JCD, Macaulay JDJ, Mcconnell BJ, Sparling CE. Tracking Technologies for Quantifying Marine Mammal Interactions with Tidal Turbines: Pitfalls and Possibilities In: Shields MA, Payne AIL, editors. Marine Renewable Energy Technology and Environmental Interactions, Humanity and the Sea; 2014 p. 127–139.

[7] Benjamins S, Dale AC, Hastie G, Waggitt JJ, Lea MA, Scott B, Wilson B. Confusion Reigns? A Review of Marine Megafauna Interactions with Tidal-Stream Environments Oceanography and Marine Biology. 8 2015; 1–54

[8] MacAulay JDJ, Gordon JCD, Gillespie DM, Malinka CE, Northridge SP. Passive acoustic methods for fine-scale tracking of harbour porpoises in tidal rapids. Journal of the Acoustical Society of America. 2017 2;141(2):1120–1132. 10.1121/1.4976077 28253702

[9] Viehman HA, Zydlewski GB. Fish Interactions with a Commercial-Scale Tidal Energy Device in the Natural Environment. Estuaries and Coasts. 2014; p. 1–12.

[10] Lieber L, Williamson B, Jones CS, Noble LR, Brierley A, Miller P, et al. Introducing novel uses of multibeam sonar to study basking sharks in the light of marine renewable energy extraction. In: Proceedings of the 2nd International Conference on Environmental Interactions of Marine Renewable Energy Technologies; 2014.

[11] Williamson BJab, Blondel Pb, Armstrong Ec, Bell PSd, Hall Cc, Waggitt JJa, et al A Self-Contained Subsea Platform for Acoustic Monitoring of the Environment Around Marine Renewable Energy Devices-Field Deployments at Wave and Tidal Energy Sites in Orkney, Scotland. IEEE Journal of Oceanic Engineering. 2016;41(1):67–81. 10.1109/JOE.2015.2410851

[12] Shen H, Zydlewski GB, Viehman HA, Staines G. Estimating the probability of fish encountering a marine hydrokinetic device. Renewable Energy. 2016;97:746–756. 10.1016/j.renene.2016.06.026

[13] Bevelhimer M, Colby J, Adonizio MA, Tomichek C, Scherelis C. Informing a Tidal Turbine Strike Probability Model through Characterization of Fish Behavioral Response using Multibeam Sonar Output. Oak Ridge National Laboratory; 2016 7 Available from: http://www.ntis.gov/help/ordermethods.aspx$\delimiter″026E30F$nhttp://www.osti.gov/contact.html.

[14] Cotter E, Matzner S, Horne J, Murphy P. Benchmarking Sensor Fusion Capabilities of an Integrated Monitoring Package. In: Paper Presented at the 4th Marine Energy Technology Symposium (METS); 2016.

[15] Hammar L, Eggertsen L, Andersson S, Ehnberg J, Arvidsson R, Gullstrm M, et al A probabilistic model for hydrokinetic turbine collision risks: Exploring impacts on fish. PLoS ONE. 2015;10(3). 10.1371/journal.pone.0117756 PMC4346259 25730314

[16] Schweizer PE, Cada GF, Bevelhimer MS. Estimation of the Risks of Collision or Strike to Freshwater Aquatic Organisms Resulting from Operation of Instream Hydrokinetic Turbines. Oak Ridge National Laboratory; 2011. ORNL/TM-2011/133.

[17] Whittaker T, Folley M. Nearshore oscillating wave surge converters and the development of Oyster. Philosophical Transactions of the Royal Society A: Mathematical, Physical and Engineering Sciences. 2012;370(1959):345–364. 10.1098/rsta.2011.0152 22184665

[18] Wilkinson L, Doherty K, Henry A, Russo V, Day S, Whittaker T. Wave loads on the foundation of a bottom-hinged modular flap structure. In: International Conference on Offshore Renewable Energy; 2014.

[19] Forbush D, Polagye B, Thomson J, Kilcher L, Donegan J, McEntee J. Performance characterization of a cross-flow hydrokinetic turbine in sheared inflow. International Journal of Marine Energy. 2016;16:150—161. 10.1016/j.ijome.2016.06.001.

[20] Zambrano C. Lessons learned from subsea tidal kite quarter scale ocean trials In: WTE16—Second Workshop on Wave and Tidal Energy. Valdivia, Chile; 2016.

[21] Alarcón-Rodrigues JR, Martínez-Fadrique FM, Klinkrad H. Development of a collision risk assessment tool. Advances in Space Research. 2004;34(5):1120–1124. 10.1016/j.asr.2003.01.015

[22] Lidtke AA, Lewis HG, Armellin R, Urrutxua H. Considering the collision probability of Active Debris Removal missions. Acta Astronautica. 2017;131:10—17. 10.1016/j.actaastro.2016.11.012.

[23] Bai XZ, Ma CW, Chen L, Tang GJ. Maximum collision probability considering variable size, shape, and orientation of covariance ellipse. Advances in Space Research. 2016;58(6):950—966. 10.1016/j.asr.2016.05.050.

[24] Lin M, Xu M, Fu X. A Parallel Algorithm for the Initial Screening of Space Debris Collisions Prediction using the SGP4/SDP4 models and {GPU} Acceleration. Advances in Space Research. 2017; p. –. 10.1016/j.asr.2017.02.023.

[25] Curtis RG. A ship collision model for overtaking. Journal of the Operational Research Society. 1986;37(4):397–406. 10.1057/jors.1986.67

[26] Bocci F. Whether or not to run in the rain. European Journal of Physics. 2012;33(5):1321 10.1088/0143-0807/33/5/1321

[27] Copping A, Grear M, Jepsen R, Chartrand C, Gorton R. Understanding the potential risk to marine mammals from collision with tidal turbines. International Journal of Marine Energy. 2017;19:110–123. 10.1016/j.ijome.2017.07.004

[28] Available from: https://www.gitbook.com/book/yorikvanhavre/a-freecad-manual.

[29] R Core Team (2016) R: a language and environment for statistical computing. R Foundation for Statistical Computing, Vienna, Austria https://www.R-project.org.

